# Corneal Sensitivity to Hyperosmolar Eye Drops: A Novel Behavioral Assay to Assess Diabetic Peripheral Neuropathy

**DOI:** 10.1167/iovs.16-19435

**Published:** 2016-05-04

**Authors:** Matthew S. Yorek, Eric P. Davidson, Pieter Poolman, Lawrence J. Coppey, Alexander Obrosov, Amey Holmes, Randy H. Kardon, Mark A. Yorek

**Affiliations:** 1 Department of Veterans Affairs Iowa City Health Care System, Iowa City, Iowa, United States; 2 Veterans Affairs Center for the Prevention and Treatment of Visual Loss, Iowa City, Iowa, United States; 3 Department of Internal Medicine, University of Iowa, Iowa City, Iowa, United States; 4 Department of Ophthalmology and Visual Sciences, University of Iowa, Iowa City, Iowa, United States; 5 Fraternal Order of Eagles Diabetes Research Center, University of Iowa, Iowa City, Iowa, United States

**Keywords:** diabetes, diabetic neuropathy, cornea, corneal confocal microscopy, hyperosmolarity

## Abstract

**Purpose:**

Diagnosis of peripheral neuropathy (PN), which affects approximately 50% of the diabetic population, is subjective, with many patients seeking a diagnosis only after presenting with symptoms. Recently, in vivo confocal microscopy of subepithelial corneal nerve density has been promoted as a surrogate marker for early detection of PN, but imaging of corneal nerves requires sophisticated instrumentation, expertise in confocal imaging, cooperative patients, and automated analysis tools to derive corneal nerve density. As an alternative, we developed a simple screening method that is based on the sensitivity of corneal nerves to cause reflex eyelid squinting in response to hyperosmolar eye drops.

**Methods:**

Eyes of control and type 2 diabetic rats were given an eye drop of a 290- to 900-mOsm solution, and the ocular response was video recorded. Other neuropathic end points including nerve conduction velocity and subepithelial cornea nerve density were determined.

**Results:**

Motor and sensory nerve conduction velocity and total nerve fiber length of corneal nerves in the subepithelial layer were significantly decreased in diabetic rats. Applying the hyperosmotic solutions to the ocular surface caused an osmolarity-dependent increase in squinting of the treated eye in control rats. Squinting was almost totally blocked by preapplication of proparacaine or *N*-(4-tertiarybutylphenyl)-4-(3-chloropyridin-2-yl)tetrahydropyrazine-1(2H)-carbox-amide, a transient receptor potential melastatin-8 channel blocker. Squinting in response to the 900-mOsm solution was significantly reduced in diabetic rats.

**Conclusions:**

Preclinical studies show that evaluation of corneal sensitivity may be an alternative method for the early detection of PN and has potential for translation to clinical studies.

Diabetic peripheral neuropathy (PN) is a common complication of diabetes, with no known treatment besides normalization of blood glucose.^1^ Should a treatment for diabetic PN become available, early detection will become a key to improving outcome. Presently, the clinical diagnosis for diabetic PN is subjective, with many patients seeking a diagnosis only after presenting with symptoms of advanced PN. Earlier diagnosis of PN is needed if new treatments aimed at preserving nerves and stimulating regeneration is to be successful. Recently, determining subepithelial corneal nerve density has been promoted as a surrogate marker for early diagnosis of diabetic PN.^2–5^ In vivo imaging of corneal nerves by corneal confocal microscopy is a noninvasive procedure, but it can be challenging to implement as a routine examination. Moreover, many images must be acquired and made into a montage for analysis, which can be very labor intensive. As an alternative, we developed an objective functional test of corneal sensitivity.^6^ The method uses hyperosmolar eye drops to activate transient receptor channel-8 receptors (TRPM8) in the cornea, which results in reflex blinking and squinting if the nerves are intact.^7^ As diabetic PN develops, damaged corneal nerves lose sensation, which decreases the reflex response to corneal stimulation.^8^ The motivation for this study was to develop a method of early detection of PN that can be performed annually during a routine clinical visit or eye examination and that could be used to monitor treatment to greatly improve the standard of care for diabetic patients.

## Methods

Unless stated otherwise, all chemicals used in these studies were obtained from Sigma-Aldrich Corp. (St. Louis, MO, USA).

### Animals

Male Sprague-Dawley (Harlan Sprague Dawley, Indianapolis, IN, USA) rats of 10 to 11 weeks of age were housed in a certified animal care facility, and food (#7001; Harlan Teklad, Madison, WI, USA) and water were provided ad libitum. All institutional ([Animal Component of Research Protocol] ACORP #1590601) and National Institutes of Health guidelines for use of animals were followed. The studies also adhered to the ARVO statement for the use of animals in research. At 12 weeks of age, rats were separated into two groups. One group was placed on a high-fat diet (D12451 [45% kcal as fat, 4.7 kcal/g]; Research Diets, New Brunswick, NJ, USA). The high-fat diet contained 24 g% fat, 24 g% protein, and 41 g% carbohydrate. The primary source of the increased fat content in the diet was lard. The other group was maintained on the control diet (#7001, 3.0 kcal/g; Harlan Teklad), which contained 4.25 g% fat. Rats were maintained on these diets for 8 weeks. Afterward, the high-fat–fed rats were treated with streptozotocin (30 mg/kg in 0.1 M citric acid buffer, pH 4.5, intraperitoneally [IP]; EMD/Millipore, Billerica, MA, USA). Diabetes was verified 96 hours later by evaluating blood glucose levels with the use of glucose-oxidase reagent strips (Aviva Accu-Chek; Roche, Mannheim, Germany). Rats having a blood glucose level of 250 mg/dL (13.8 mM) or greater were considered to be diabetic. Control and diabetic rats were maintained on their respective diets, and 18 weeks later, analyses were performed.

### Glucose Tolerance

Glucose tolerance was determined by injecting rats with a saline solution containing 2 g/kg glucose, IP, after an overnight fast as previously described.^9^

### Thermal Nociceptive Response and Corneal Sensitivity

Thermal nociceptive response in the hindpaw was measured using the Hargreaves method as previously described.^10^ Data were reported in seconds. Tactile responses were evaluated by quantifying the withdrawal threshold of the hindpaw in response to stimulation with flexible von Frey filaments as previously described.^11^ Data were reported in grams. Corneal sensation was measured using a Cochet-Bonnet filament esthesiometer in unanaesthetized rats (Luneau Ophtalmogie, Prunay-le-Gillon, France) as previously described.^12^ The data were reported in centimeters of filament length.

Corneal sensation was also measured by applying buffered hyperosmotic eye drops (290–900 mOsm/L), Muro-128 solution 2% sodium chloride (684 mOsm/L), or 5% sodium chloride (1710 mOsm/L) to unanaesthetized rats. We also examined the ability of proparacaine (local anesthetic) or *N*-(4-*tert*-butylphenyl)-4-(3-chloropyridin-2-yl)piperazine-1-caboxamide (BCTC; TRPM8 blocker) to inhibit the squinting effect induced by a 900-mOsm solution.^7^ For this study, prior to the addition of the hyperosmotic solution, a drop of proparacaine hydrochloride ophthalmic solution, 0.5% (Sandoz, Inc., Princeton, NJ, USA), or 20 μL BCTC, 50 μM, (Tocris, Bristol, United Kingdom) was applied to the ocular surface. These studies were performed by placing conditioned animals in a custom-made restraining apparatus and allowing 5 minutes for the animal to acclimate to the restraint device and lighting. Three CMOS cameras (Imaging Development Systems GmbH, Obersulm, Germany), one positioned in front of the animal and two positioned laterally, were used to observe both eyes simultaneously. Custom software was used to synchronize video streams and obtain images (MATLAB R2012a; The MathWorks, Inc., Natick, MA, USA). Video was taken continuously at 30 fps of each animal as an isotonic solution, and increasing concentrations of hyperosmolar solutions were delivered to the left eye. The osmolarity of the pH 7.4 PBS solutions used for these studies was 290 (isotonic), 375, 550, 725, and 900 mOsm. Osmolarity of the buffer was adjusted using sucrose. Temperature of the solutions was maintained at a 25 ± 1°C. During each recording epoch, 20 μL solution was instilled in the left eye, and the response was video recorded for 150 seconds after a 10-second delay. An image collector was used offline to retrieve video frames every 10 seconds from each eye drop concentration epoch; therefore, 15 images were analyzed per animal per concentration. Fiji-image J image analysis software was used by a masked technician to measure the visible surface area of both eyes between the upper and lower eyelids.^13^ In each recording epoch, the values obtained are presented as a percentage of the animals' maximum visible eye surface area between the eyelids during that epoch.

### Corneal Innervation

On the day of the terminal studies, rats were weighed and anesthetized with Nembutal i.p. (50 mg/kg, IP; Abbott Laboratories, North Chicago, IL, USA). Subepithelial corneal nerves were imaged using the Rostock cornea module of the Heidelberg Retina Tomograph confocal microscope as previously described.^12^ The investigator acquiring these images was masked with respect to identity of the animal condition. Corneal nerve fiber length was defined as the total length of all nerve fibers and branches (in millimeters) present in the acquired images standardized for area of the image (in square millimeters).^12^ The corneal fiber length for each animal was the mean value obtained from the acquired images and expressed as mm/mm^2^. Based on receiver operating characteristic (ROC) curve analysis, corneal nerve fiber length is the optimal parameter for diagnosing patients with diabetic neuropathy and has the lowest coefficient of variation.^14,15^

After corneal confocal microscopy, corneas were dissected from the eyes and trimmed around the sclero-limbo region. Corneas were fixed with Zambonis (American MasterTech Scientific, Inc., Lodi, CA, USA) for 30 minutes, permeabilized using 5% Triton X-100 for 1 hour, blocked in 2% goat serum and 1% bovine serum albumin for 2 hours, and then incubated with mouse monoclonal neuronal class III β-tubulin 1:1000 and TRPM8 antibody^16^ 1:1000 in blocking buffer overnight at 4°C (Covance, Dedham, MA, USA). After washing with incubation buffer, the tissue was incubated with Alexa Flour 488 goat anti-mouse IgG_2a_ and Alexa Fluor 546 goat anti-rabbit IgG 1:500 in blocking buffer for 2 hours at room temperature (Invitrogen, Eugene, OR, USA). After washing, the cornea was placed epithelium side up on a microscope slide. Excess water was carefully aspirated, and five radial cuts were made. The tissue was carefully covered with a cover slip, mounted with ProLong Gold (Thermo Fisher Scientific, Waltham, MA, USA), and sealed with clear nail polish. A 3x2 matrix tile scan and Z-stack images were collected using a Zeiss LM710 confocal microscope with ZEN software (Carl Zeiss Microscopy, Thornwood, NY, USA). Images were prepared using Imaris and Fiji software for presentation (Bitplane, Zurich, Switzerland).

### Motor and Sensory Nerve Conduction Velocity

Motor nerve conduction velocity was determined as previously described using a noninvasive procedure in the sciatic-posterior tibial conducting system.^10^ Sensory nerve conduction velocity was determined using the digital nerve as described by Obrosova et al.^17^ Motor and sensory nerve conduction velocities were reported in meters per second.

### Intraepidermal Nerve Fiber Density in the Hindpaw

Immunoreactive nerve fiber profiles innervating the skin from the hindpaw were visualized using standard confocal microscopy as previously described.^10,12^ Profiles were counted by two individual investigators that were masked to the sample identity. All immunoreactive profiles were counted and normalized to length, and the data are presented as profiles per millimeter.

### Physiological Markers

Additional measurements taken included nonfasting blood glucose and hemoglobin A_1_C levels (Glyco-tek affinity column; Helena Laboratories, Beaumont, TX, USA). Serum was collected for determining levels of free fatty acid, triglyceride, and free cholesterol using commercial kits from Roche Diagnostics; Sigma-Aldrich Corp.; and Bio Vision (Mountain View, CA, USA), respectively. Liver steatosis was determined as previously described.^10^ Acquired images were analyzed for % area fraction of lipid droplets using Image J software (http://imagej.nih.gov/ij/; provided in the public domain by the National Institutes of Health, Bethesda, MD, USA).

### Data Analysis

Results are presented as mean ± SEM. Comparisons between the control and diabetic rats were conducted using Student's *t*-test (Prism software; GraphPad, San Diego, CA, USA). Correlation coefficients analyses were determined using Prism software. *P* < 0.05 was considered significant.

## Results

**Figure 1:**
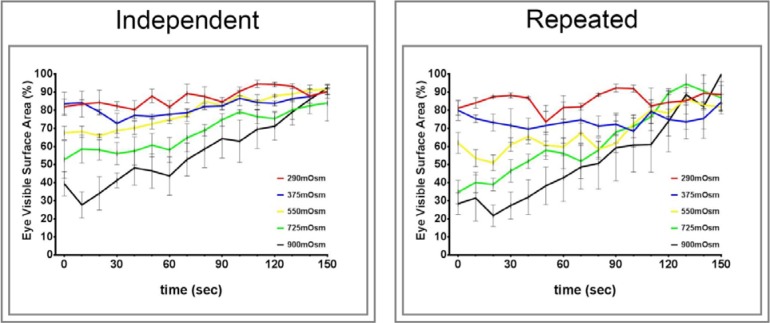
Effect of application of isotonic and increasing hypertonic eye drop on eye closure (squinting) in normal Sprague-Dawley rats. Two protocols were used to access the effect of hyperosmolarity on squinting, which was examined as described in the Methods section. The graph on the right “Independent” used four untested rats for each different osmotic solution. The graph on the left “Repeated” used the same four rats with a 2-day rest between each application. For the latter group, the isotonic, 290-mOsm solution was applied first followed by increasing osmolarity with each application. Data are presented as the mean ± SEM for % visible surface area of the eye.

Figure 1 provides data of the effect of increasing osmolarity on squinting by control rats. For the graph labeled “Independent,” data were acquired using four different rats for each osmotic solution. For the graph labeled “Repeated,” data were acquired using the same four rats with 2-day rest between applications of each osmotic solution. Both protocols demonstrated that increasing the osmolarity of the buffer from an isotonic osmolarity of 290 to 900 mOsm increased squinting of the treated eye and that the amount of squinting was dependent on the osmolarity. Recovery from the 900 mOsm solution was complete in approximately 150 seconds. In contrast, no change in eye closure was observed in the untreated eye (data not shown).

**Figure 2:**
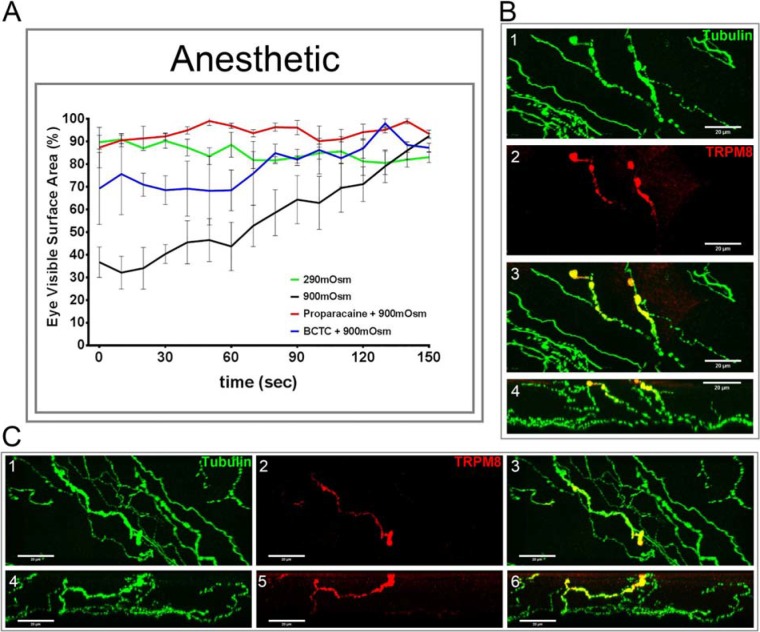
Effect of proparacaine or BCTC on eye closure (squinting) in response to an application of 900-mOsm solution in normal Sprague-Dawley rats. Squinting in response to an eye drop of a 290- or 900-mOsm solution was examined as described in the Methods section (**A**). For this study, we also examined the effect of proparacaine or BCTC on hyperosmotic effect. Five and 10 minutes prior to the addition of the hyperosmotic solution (900 mOsm), a drop of proparacaine hydrochloride ophthalmic solution, 0.5%, or 20 μL 50 μM BCTC was applied to the ocular surface. Data are presented as the mean ± SEM for % visible surface area of the eye. Four animals were examined. In **B1**–**B3** and **C1**–**C3** (maximum projection) and **B4** and **C4**–**C6** (axial projection), representative images are provided for immunostaining of corneal nerves with antibodies to neuronal class III tubulin (*green* [**B1**, **C1**]) and TRPM8 (*red* [**B2**, **C2**]). A merge imaged for each of the two examples are shown (**B3**, **C3**). In **B**, the fourth image is an axial projection of the epithelial layer of the cornea. **B** shows two single simple filaments penetrating the epithelial layers, whereas **C** demonstrates a bifurcating nerve with in the epithelium.

Data in Figure 2A demonstrate that the effect of the 900-mOsm solution on squinting by control rats was almost totally prevented by the anesthetic proparacaine and by BCTC and TRPM8 channel blocker. Data in Figures 2B, 2C present corneal epithelial nerves from a control rat immunostained with antibodies to neuronal class III β-tubulin and TRPM8.^16^ These data clearly demonstrate that some corneal nerves in the subepithelial layer and penetrating the cornea epithelium express TRPM8 channels.

**Figure 3:**
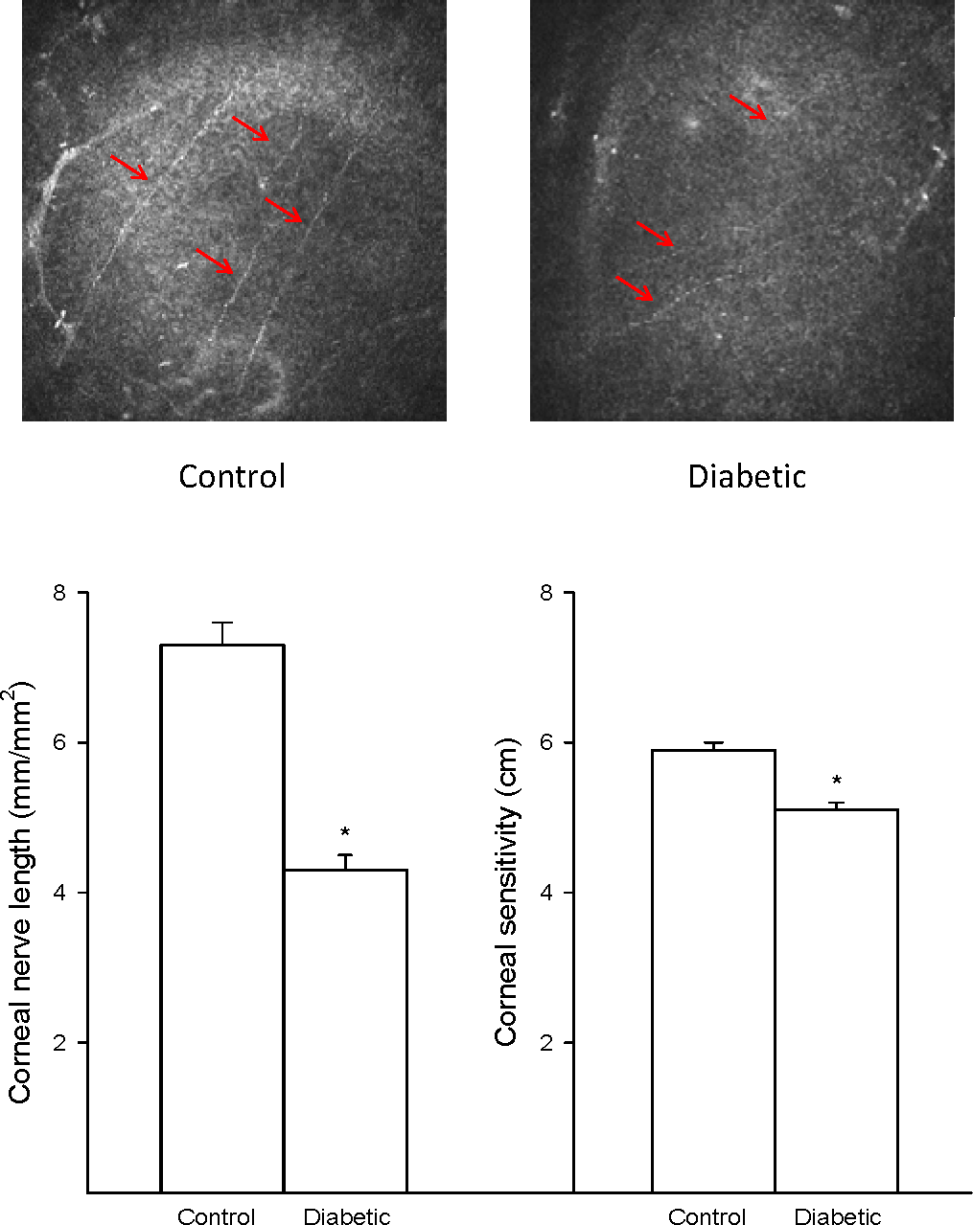
Effect of type 2 diabetes induced by high-fat diet and low-dose streptozotocin in Sprague-Dawley rats on innervation of the subbasal layer of the cornea and corneal sensitivity. Innervation of the subbasal layer of the cornea was determined by using corneal confocal microscopy as described in the Methods section. Corneal sensitivity was determined by using a Cochet-Bonnet filament esthesiometer as described in the Methods section. The number of rats in each group was the same as described in Table 1. Data are presented as the mean ± SEM for innervation of the cornea in mm/mm^2^ cm for corneal sensitivity. *P < 0.05 compared with control rats.

To investigate whether corneal nerve sensitivity to a hyperosmotic solution is reduced by diabetes, we fed Sprague-Dawley rats a high-fat diet for 8 weeks and then treated them with a low dose of streptozotocin. Eighteen weeks after treatment with streptozotocin and onset of hyperglycemia, examinations were performed. The diabetic rat created by this protocol models late-stage type 2 diabetes, and we previously characterized the peripheral neuropathy in this model.^10^ The data in Table 1 and Supplementary Figures S1 and S2 confirm the presence of diabetes and PN. Diabetic rats had impaired glucose tolerance, hyperglycemia and hyperlipidemia, fatty liver, slowing of motor and sensory nerve conduction velocities, hypoalgesia with tactile allodynia, impaired vascular reactivity of epineurial arterioles to the sciatic nerve, and decreased intraepidermal nerve fibers in the skin. In addition, data in Figure 3 demonstrate that diabetic rats have a decrease in corneal nerves of the subepithelial layer and reduced cornea mechanical sensitivity as determined using a Cochet-Bonnet filament esthesiometer.

**Table 1:**
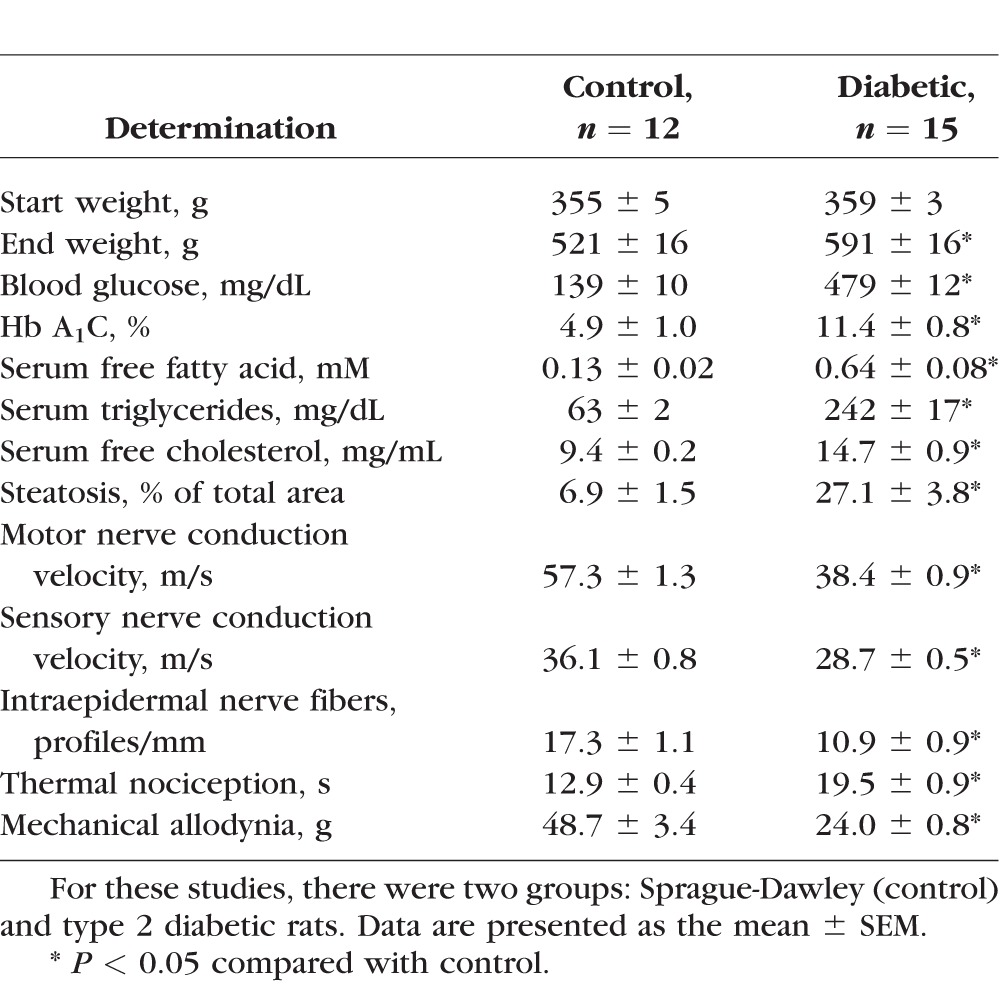
Vital Parameters for Sprague-Dawley Control and Type 2 Diabetic Rats

**Figure 4:**
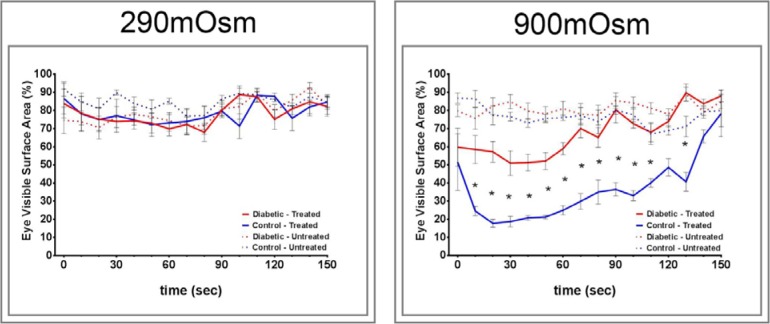
Effect of type 2 diabetes induced by high-fat diet and low-dose streptozotocin in Sprague-Dawley rats on eye closure (squinting) in response to an application of 290- and 900-mOsm solution in the treated and untreated eye. Squinting in response to an eye drop of a 290-and 900-mOsm solution was examined as described in the Methods section. The number of rats in each group was the same as described in Table 1. Data are presented as the mean ± SEM for % visible surface area of the eye. *P < 0.05 compared with control rats.

Data in Figure 4 demonstrate that corneal sensitivity to application of a 900-mOsm solution was significantly decreased in type 2 diabetic rats compared with control rats as determined by the reduced squinting of the treated eye. Control rats in this study did not fully recover from the 900-mOsm solution after 150 seconds, indicating a continuing adverse effect to the hyperosmotic solution that has not completely resolved. Neither control nor diabetic rats showed a change in eye closure in response to application of a 290-mOsm solution. The unstimulated right eye of control and diabetic rats did not respond when a drop of 290- or 900-mOsm solutions was administered to the left eye. There was a significant correlation between corneal sensitivity in response to a hyperosmotic solution to corneal nerve fiber length, corneal sensitivity as measured by the Cochet-Bonnet filament esthesiometer, intraepidermal nerve fiber density, thermal nociception, and motor and sensory nerve conduction velocities (Table 2).

**Table 2:**
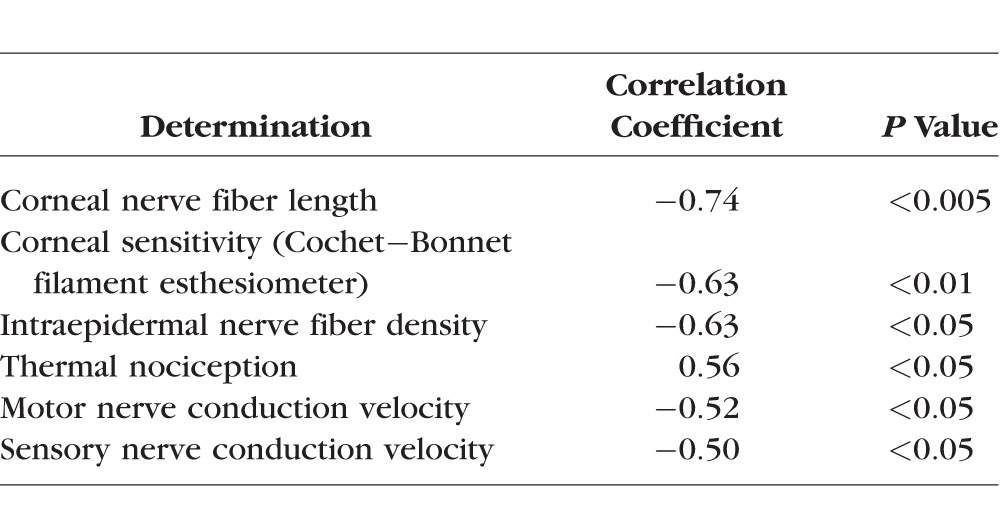
Correlation Coefficients for Corneal Sensitivity to Hyperosmotic Eye Drops (Eye Visible Surface Area) and Other Corneal and Neuropathic End Points

**Figure 5:**
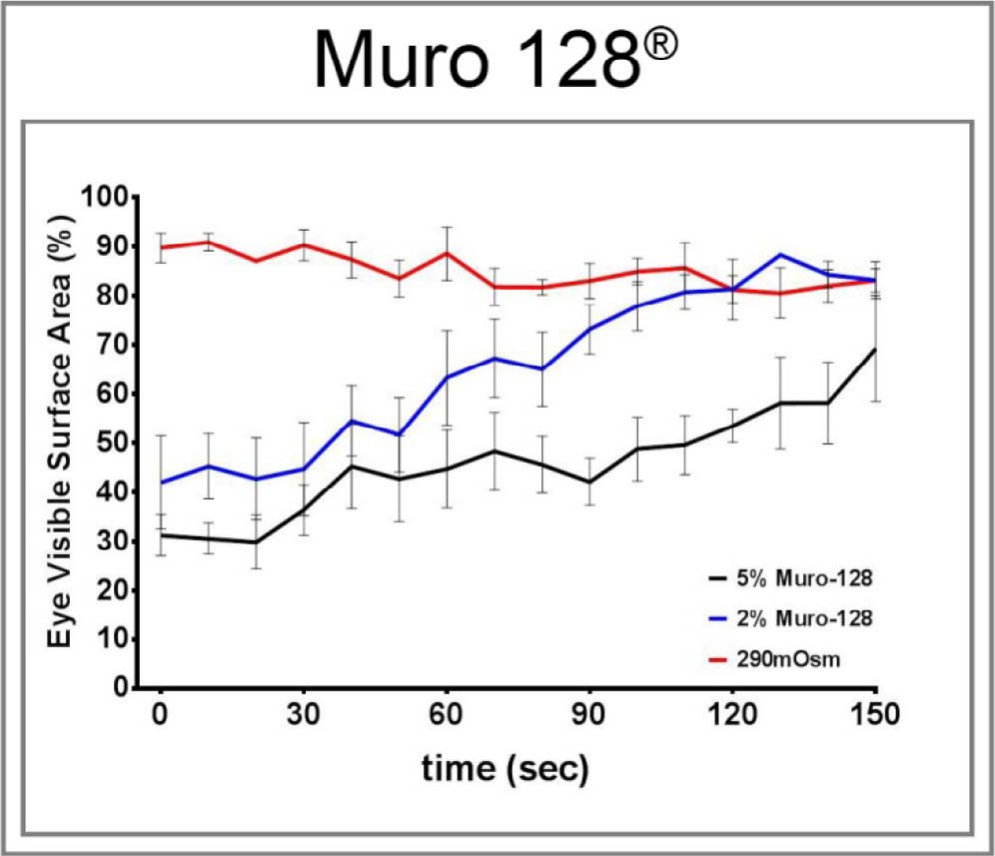
Effect of a 290-mOsm solution and 2% and 5% Muro 128 on eye closure (squinting) in normal Sprague-Dawley rats. Squinting in response to an eye drop of a 900-mOsm solution and 2% and 5% Muro 128 was examined as described in the Methods section. Data are presented as the mean ± SEM for % visible surface area of the eye.

In anticipation of applying this sensitivity assay to human subjects, we tested the corneal sensitivity of control rats to 2% and 5% Muro 128, an over-the-counter product designed for human use. Muro 128 is used clinically to reduce swelling of the surface of the eye (cornea) in certain eye conditions. It is a hypertonic solution that works by drawing fluid out of the cornea to reduce swelling. We calculated the hypertonicity of 2% and 5% Muro 128 to be 684 and 1711 mOsm, respectively. Data in Figure 5 demonstrate that both 2% and 5% Muro 128 cause squinting by control rats, with 5% Muro 128 having a greater effect than 2% Muro 128. The effect of 2% Muro 128 was similar to the effect of the 725-mOsm solution (Fig. 1). The maximum effect of 5% Muro 128 on squinting was similar to the 900-mOsm solution (Fig. 1). However, unlike the 2% Muro 128 or 900-mOsm solution, rats did not fully recover from the application of 5% Muro 128 after 150 seconds.

## Discussion

In this study, we investigated an alternative method for detecting diabetic PN using corneal sensitivity to hyperosmolar eye drops. It has been demonstrated in both human and animal subjects that diabetes alters the structure of corneal nerves, and this may be an early marker for diabetic PN.^2–6,12,18–25^ We propose that a diabetes-induced loss in corneal nerve density will translate into a decrease in cornea sensitivity in response to corneal stimulation through hypertonicity that can be evaluated by capturing and measuring behavioral changes in facial and/or ocular movement. Quantifying these behavioral changes could provide an objective screening tool for detection of PN that can be easily performed during a routine eye examination.

The major findings from this study were that exposing rat cornea to a hyperosmotic solution induces a squinting-like response that occurs only in the treated eye, is dependent on the hypertonicity of the solution, is transient, is reproducible, and is almost completely blocked by pretreatment with topical proparacaine or BCTC. This behavioral response was significantly diminished in a rat model of type 2 diabetes and showed significant correlation to a number of diabetes-induced functional and structural end points of PN.

Eyelid squint is accomplished by contracting the orbicularis oculi muscle, which completely encircles each eye. The palpebral portion of the orbicularis muscle extends through the eyelids and is responsible for blinking of the eyes. The orbital portion of the orbicularis muscle encircles the eye more peripherally and extends above the brow and even below the zygomatic process. Contraction of the orbital portion of the orbicularis draws the brows and cheeks closer to the eyes and results in eyelid squint with narrowing of the palpebral fissure.^26^ Squinting is commonly associated with photophobia and the trigeminal tactile corneal blink reflex in humans and can also improve visual acuity through the pinhole effect of a narrow slit.^26,27^ However, our studies demonstrate that squinting is also associated with activation of osmolarity sensing channels in the cornea of the rat. Rahman et al.^28^ show that hypertonic saline applied to the ocular surface of the rat elicits orbicularis muscle activity, which is dose dependent and is eliminated by corneal anesthesia, as well as by the blockade of trigeminal ganglion neurons. Recently, in the mouse, activation of TRPM8 channels in the cornea by increasing osmolarity was associated with an increased rate of blinking.^7^ We found that the rat displays a low rate of blinking and that hyperosmolar activation of TRMP8 channels was associated with increased squinting rather than blinking. In this study, changes in squinting, with narrowing of the palpebral fissure in response to activation of the neuronal osmosensor channels, were used to examine the effect of loss of neuronal sensation caused by diabetes.^7,8^ In human studies, Pritchard et al.^6^ use noncontact corneal esthesiometry to examine corneal sensitivity in subjects with type 2 diabetes. This test requires the subject to verbally indicate whether they felt the sensation from increasing amounts of pressure introduced from a nozzle 10 mm from the center of the cornea.^6^ They found noncontact corneal esthesiometry to be a sensitive test for the diagnosis of minimal and more advanced diabetic neuropathy, and it may serve as a useful surrogate marker. The noncontact corneal esthesiometry test measures the mechano-sensitivity of the corneal nerves, whereas our test using hyperosmotic solutions is likely to be more specific for the TRPM8-expressiong sensory neurons.^7^ Noncontact corneal esthesiometry also requires a verbal confirmation by the subject of whether they felt the stimulus. Our test measures a physical response to the stimulus (orbicularis oculi activation) and thus may be more reliable.

Sensations evoked at the ocular surface arise from the activation of several functional classes of primary sensory neurons located in the trigeminal ganglion. Based on their response to specific stimuli, sensory nerve fibers identified in the cornea include Aδ mechanonociceptors (∼20% of fibers) that react only to mechanical forces; polymodal nociceptors (∼70% of fibers) that respond to mechanical forces but also to heat, exogenous chemical irritants, and endogenous inflammatory mediators; and C fiber cold-sensitive receptors (∼10% of fibers) that react to cooling.^8,29^ Collectively, these fiber types have been implicated in animal models of neuropathic pain, possibly via expression of transient receptor potential (TRP) family members such as TRP vanilloid receptor (TRPV1), transient receptor potential ankyrin 1 (TRPA1), TRPM8, and Piezo2.^29,30^ Pertinent to our study are expression and sensitivity of TRPM8 because Quallo et al.^7^ demonstrated that TRPM8 is a neuronal osmo-sensor that regulates blinking in mice. Alamri et al.^31^ demonstrated that approximately 45% of corneal afferent neurons expressed TRPV1, 28% expressed Piezo2 (a marker of putative pure mechano-nociceptors), and 8% expressed TRPM8, with 6% of TRPV1 neurons coexpressing TRPM8 (a marker for of cold-sensing neurons); TRPM8 cold-sensing neurons are activated by menthol and hyperosmotic stimuli.^7,32^ Our studies confirm that sensory nerves in the subepithelial layer and nerves penetrating the epithelial layer of the cornea express TRPM8. Furthermore, we found that pretreating the ocular surface with 0.5% proparacaine (a rapid acting local anesthetic) or BCTC, a blocker of menthol-evoked and cold-activated responses in TRPM8 channels, inhibited the hyperosmotic response in rats treated with a 900-mOsm solution.^33^ This suggests that increased squinting in the rat in response to exposure of the ocular surface to a hyperosmotic solution occurs, at least in part, through activation of TRPM8 channels. Our studies also demonstrate that in type 2 diabetic rats, this osmotic sensing response is impaired. Correlation coefficient analysis revealed that the osmotic sensing response in the rat correlated with other diabetes-induced end points indicative of diabetic PN. At this time we do not know if expression of TRPM8 channels in the corneal nerves is specifically decreased in cornea of diabetic rats or if the diminished response in diabetic rats to hyperosmolarity is due to the overall decrease in corneal nerves that occurs. An answer to this question will require further investigation. However, in humans, it has been shown that corneal sensitivity to mechanical, chemical, and thermal stimulation is decreased in diabetes patients, suggesting that diabetes affects homogeneously the different types of sensory neurons innervating the cornea.^8^ Future studies will also investigate whether the osmo-sensing response can be used as a marker of early detection or screening tool for diabetic PN in humans.

We also found that applying 2% or 5% Muro 128 to the ocular surface of rats resulted in a squinting response similar to the 725- and 900-mOsm solutions, respectively, with the exception that recovery from the application of 5% Muro 128 was not complete after 2.5 minutes. The calculated osmolarity of 2% and 5% Muro 128 was 684 and 1711 mOsm, respectively. The slower recovery of the squinting response observed with 5% Muro 128 compared with the 900-mOsm solution could be due to the higher osmolarity of the 5% Muro 128 solution or the difference between sodium chloride and sucrose on the sensory channel, independent of osmolarity. Because Muro 128 is a product produced commercially for human use to reduce swelling of the surface of the eye (cornea) in certain eye conditions, adapting it as the hyperosmotic solution to be used in human studies pertaining to cornea sensitivity in diabetic subjects with or without neuropathy is a viable testing approach.

In a pilot study that we have already begun in human subjects, the Muro 128 5% NaCl drop had been well tolerated, causing only mild discomfort (less than from a mydriatic drop), and results in a transient increase in blink rate and squinting in normal eyes during the first 60 seconds after administration. Muro128 is convenient to use, widely available in an over-the-counter preparation, and is commonly used for treating corneal edema. We have been developing a completely automated portable video-based system for recording and analysis of blink rate and squinting in both animals and humans, based on real-time image analysis of facial features recorded from a multicamera video system. Our intent is to make available an easy to use, fully automated, portable system for assessing corneal sensitivity in the context of diabetic PN screening that can be performed by noneye care specialists. The study reported here in rats is in parallel with human studies in progress and would be the first reported study to validate the osmotic reflex blinking/squinting response in a rodent model of progressive diabetic PN. Therefore, the present study has significant importance because it is translatable to human assessment of PN and shows that this simple test correlates with other measures of PN.

In summary, this study has shown that osmotic sensing of the cornea is impaired in rats with chronic type 2 diabetes. Further studies will determine whether this behavioral response can be used as an early marker for diabetic PN and whether it is translational to human diabetic subjects. Development of a method of early detection of PN that can be performed annually during a routine clinical visit or eye examination and that could be used to monitor treatment would greatly improve the standard of care for diabetic patients.

## Supplementary Material

Supplement 1

Supplement 2
