# Supplementary material for: Corneal Sensitivity to Hyperosmolar Eye Drops: A Novel Behavioral Assay to Assess Diabetic Peripheral Neuropathy
Source: Invest Ophthalmol Vis Sci. 2016 May 4;57(6):2412–9. doi: 10.1167/iovs.16-19435 (PMC5113984; doi:10.1167/iovs.16-19435)
Supplement: Supplement 1 [file i1552-5783-57-6-2412-s01.pdf]

Figure 1 Supplemental

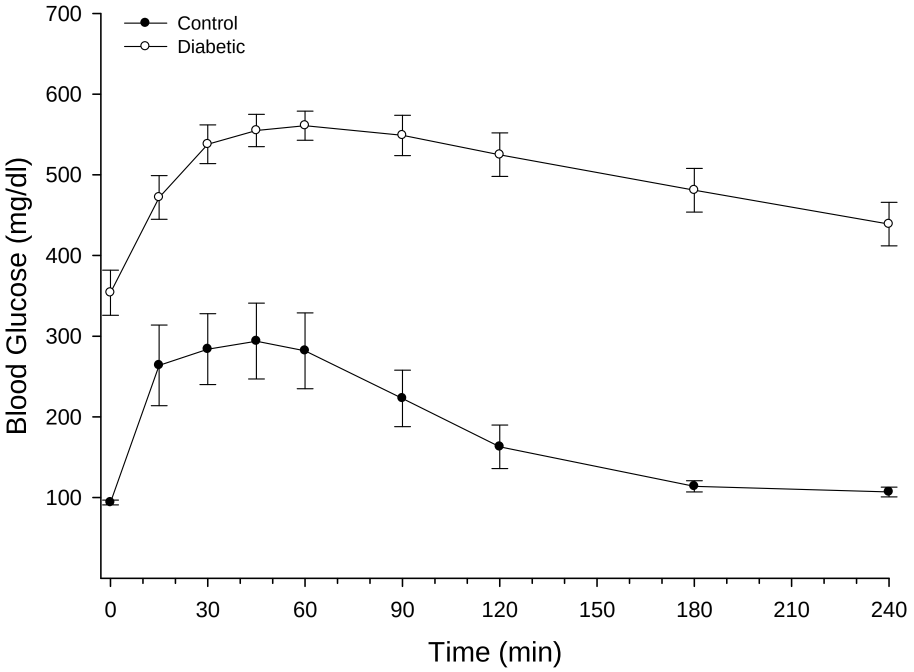

Figure 1 supplemental. Effect of type 2 diabetes induced by high fat diet and low dose streptozotocin in Sprague-Dawley rats on glucose tolerance. Glucose clearance was determined as described in the Methods section. Data are presented as the mean ± S.E.M. for glucose utilization in mg/dl. The number of rats in each group was from 12-15.
