# Supplementary material for: Corneal Sensitivity to Hyperosmolar Eye Drops: A Novel Behavioral Assay to Assess Diabetic Peripheral Neuropathy
Source: Invest Ophthalmol Vis Sci. 2016 May 4;57(6):2412–9. doi: 10.1167/iovs.16-19435 (PMC5113984; doi:10.1167/iovs.16-19435)
Supplement: Supplement 2 [file i1552-5783-57-6-2412-s02.pdf]

Figure 2 Supplemental

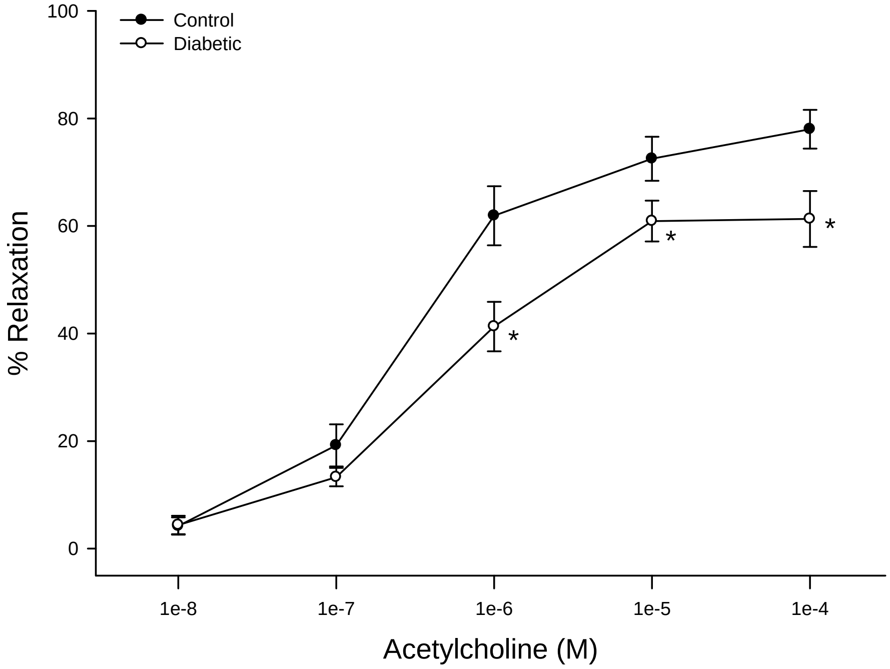

Figure 2 supplemental. Effect of type 2 diabetes induced by high fat diet and low dose streptozotocin in Sprague-Dawley rats on vascular relaxation by acetylcholine in epineurial arterioles. Pressurized arterioles (40 mm Hg and ranging from 60-100 µm luminal diameter) were pre-constricted with phenylephrine (30-50%) and incremental doses of acetylcholine were added to the bathing solution while recording steady state vessel diameter. Data are presented as the mean of % relaxation ± S.E.M. The number of rats in each group was the same as described in Table 1. * $p < 0.05$ compared to control Sprague-Dawley rats.
